# Providers’ and pregnant women’s perspectives on obstetric point-of-care ultrasound use in peri-urban Karachi, Pakistan: a qualitative study

**DOI:** 10.1186/s44247-026-00295-4

**Published:** 2026-08-20

**Authors:** Laeba Hussain, Nida Salman Yazdani, Ayesha Khalid, Fariya Ahmed, Hamna Javed, Ishrat Fatima, Imran Nisar, Fyezah Jehan, Katelyn Rittenhouse, Bethany L. Freeman, Jeffery Stringer, Zahra Hoodbhoy

**Affiliations:** 1 Department of Paediatrics and Child Health, Aga Khan University, Karachi, Pakistan; 2 Department of Obstetrics and Gynecology, University of North Carolina at Chapel Hill, Chapel Hill, NC USA

**Keywords:** Point of care ultrasound, Perceptions, Pregnant women, Healthcare providers, Low- and middle-income countries

## Abstract

**Background:**

Ultrasound is essential for antenatal care (ANC), yet access remains limited in many low- and middle-income countries (LMICs) due to shortages of equipment and trained personnel. Point-of-care ultrasound (POCUS) offers a feasible solution for gestational dating, detection of high-risk conditions, and referral decision-making in these settings. Understanding the perspectives of both healthcare providers (HCPs) and pregnant women is important for successful integration of POCUS into routine antenatal care. This study aimed to explore the perceptions of obstetric POCUS use among HCPs and pregnant women at a peri-urban community in Karachi, Pakistan.

**Methods:**

A qualitative study was conducted at a peri-urban site in Karachi, Pakistan to explore the perceptions of obstetric POCUS among HCPs and pregnant women. Purposive sampling was employed and in-depth interviews (IDIs) were conducted with pregnant women and HCPs from an ongoing POCUS cohort study until thematic saturation was achieved, yielding a total of nine participants (six pregnant women and three HCPs). Interviews were audio recorded, transcribed, translated, and analyzed using a thematic analysis framework.

**Results:**

The five common themes identified were role of ultrasound, perceived utility of POCUS, limitations and areas of improvement, myths and misconceptions, and recommendations for future use. POCUS was perceived as a valuable and feasible tool for ANC due to its portability and ability to perform rapid assessment closer to communities. Women valued reduced travel times while HCPs highlighted its user-friendly design and short learning curve for basic obstetric assessment and referral decisions. However, concerns regarding technical difficulties and diagnostic limitations were also noted. Moreover, pregnant women and providers appraised POCUS from different vantage points: women’s confidence was largely driven by the reassurance of seeing the fetus directly, while providers held a more cautious view of what the device could diagnose. Findings were consistent with key constructs of the Technology Acceptance Model (TAM), as both women and providers recognized POCUS as a practical and beneficial addition to antenatal care. Both groups supported expanding POCUS use in community-based antenatal care.

**Conclusions:**

Targeted interventions focusing on structured training and community education may support effective integration of POCUS into routine ANC and improve access to obstetric imaging in underserved communities, with the potential to improve maternal and neonatal outcomes.

**Supplementary Information:**

The online version contains supplementary material available at 10.1186/s44247-026-00295-4.

## Background

Ultrasound is a safe, non-invasive imaging modality and cornerstone of antenatal care (ANC), enabling accurate estimation of gestational age (GA), monitoring of fetal growth, assessment of placental location and amniotic fluid volume, and early detection of fetal anomalies and maternal complications [1]. The World Health Organization (WHO) recommends at least one ultrasound scan before 24 weeks of gestation to support a positive pregnancy experience [2]. However, in many low- and middle-income countries (LMICs), access to routine obstetric ultrasound remains out of reach due to shortages of trained sonologists, inadequate equipment, unreliable electricity, and high out-of-pocket costs [3]. These barriers have accelerated the adoption of point-of-care ultrasound (POCUS) as a practical, portable approach to expanding access to basic obstetric imaging.

POCUS refers to focused bedside ultrasound performed with a handheld ultrasound probe by trained health care providers (HCPs) to guide real-time clinical decision-making. First introduced nearly three decades ago, POCUS has transformed bedside medicine and evolved into a core diagnostic tool across multiple specialties worldwide, including emergency medicine, internal medicine, anesthesia, and obstetrics, that can be performed by non-specialist providers with minimal training [4]. In obstetric practice, POCUS has established itself as a vital tool for estimating GA, determining fetal presentation and viability, and assessing fetal well-being [5]. A community-based study in the Philippines reported that hand-held ultrasound performed by trained HCPs identified abnormalities in 31.7% of pregnancies and estimated that its use could avert up to 6.3% of maternal and 14.6% of neonatal deaths by enabling timely referral [6]. A Kenyan study found that the use of POCUS in rural clinics improved timely referral for high-risk pregnancies and ensured continuity of care [7]. Similarly, in rural Zambia, 21 midwives performed 441 ultrasound scans over six months, with 17% (74 cases) leading to changes in clinical decision-making [8].

Despite growing evidence of technological feasibility and operational utility, successful integration of POCUS into routine ANC depends on its acceptability and perceived value among both providers and pregnant women. Existing qualitative research has predominantly focused on providers’ experiences, often in rural or facility-based settings, and suggests that nurses and midwives view POCUS as an empowering tool that enhances clinical confidence, improves diagnostic accuracy, and facilitates timely patient referrals [9, 10]. In contrast, the perspectives of pregnant women are frequently overlooked, limiting understanding of how misalignment between providers’ and pregnant women’s perspectives could affect trust, uptake of services, and adherence to recommended ANC practices [10]. Understanding women’s perspectives is also essential for culturally appropriate use, including ensuring privacy during scans, accommodating preferences for provider gender, and addressing community beliefs about ultrasound. By examining providers’ and women’s perspectives simultaneously, this study seeks to identify barriers and facilitators to real-world implementation and to inform strategies that align clinical practice with patient expectations and cultural norms. These insights are expected to inform context-specific strategies for effective integration of POCUS into routine ANC in LMICs.

## Materials and methods

### Study design and setting

This study used an exploratory qualitative design to understand the perceptions of POCUS among pregnant women and HCPs. The study was conducted between June and September 2025 at the Primary Health Center (PHC) in Bhains Colony, a peri-urban community situated along the coastal belt of Bin Qasim Town, Karachi, Pakistan. Bhains Colony, also known as Cattle Colony, is a densely populated neighborhood with an estimated population of approximately 140,000 that has undergone rapid urbanization [11]. Female community health workers (CHWs) conduct quarterly household visits in the colony to document vital events, including pregnancies, delivery outcomes, and deaths, while also providing education on ANC, nutrition, and immunizations. In addition, trained community midwives at the PHC provide ANC services and facilitate referrals to public or private health facilities for childbirth. Maternal and newborn health data related to antenatal, delivery, and postnatal care are recorded electronically using a custom-built Android application, ensuring efficient data management and accessibility. The POCUS workflow operated parallel to this application: the Android application captured antenatal and traditional scans data recorded by the expert sonographer, while POCUS images were acquired through the study-specific app and stored on the Tricefy cloud server.

### Study participants

This qualitative study comprised two participant groups: pregnant women enrolled in the Fetal Age Machine Learning Initiative (FAMLI) cohort and HCPs directly involved in the study. FAMLI is a prospective study that enrolls pregnant women during antenatal care and follows them through delivery. POCUS scans are performed alongside a conventional ultrasound at each visit to support development and validation of GA estimation models and additional obstetric models, including fetal lie, placental location, and amniotic fluid assessment.

Pregnant women enrolled in the FAMLI cohort at the Bhains Colony site were purposively approached for participation. Eligible women were aged 15–49 years, able to communicate in Urdu, and had received at least one POCUS scan in the preceding 3 months to ensure better recall of their experiences. Pregnant women were excluded if they were unwilling or unable to provide written informed consent. Each eligible woman was contacted individually and explained the purpose of the study in detail. Those who provided written informed consent were invited for the interview; if a woman declined to consent, the next eligible participant was approached. Recruitment continued until thematic saturation was reached, defined as the point at which no new themes or meaningful insights emerged from successive interviews. HCPs who received formal POCUS training, had at least 6 months of experience performing POCUS scans, and provided written informed consent were eligible for inclusion. As part of the FAMLI study, a total of three HCPs were responsible for conducting POCUS scans for the enrolled participants, including two novice users and one expert sonologist. Given this limited and defined pool, all three were approached and consented for participation in the study.

### Data collection

Data were collected through in-depth interviews (IDIs). Semi-structured interview guides were developed through an extensive literature search aligned with study objectives (refer to S1 and S2 in Supplement for the guides). The guides included open-ended questions and probes to facilitate in-depth exploration of participants’ experiences. The guides for both groups focused on key domains stated in the Technology Acceptance Model (i.e. perceived usefulness, perceived ease of use, and behavioral intentions) [12]. Specifically, for pregnant women, we focused on their experience with POCUS, comfort, and trust in information available from the device, cultural and family beliefs, perceived benefits and limitations, and willingness for future use. The guides for HCPs explored themes related to routine antenatal care practices, experience with POCUS, perceived advantages and challenges, patient acceptance, and strategies to enhance its adoption. The IDI guides were forward- and back-translated into Urdu by two team members who were fluent in both languages. Three pilot interviews with pregnant women (p1–p3) were conducted at the study site to assess clarity and relevance; feedback was used to refine wording and add probes to the final guides. These three pilot interviews were used only for guide refinement and were not included in the analysis; the interviews analyzed for this manuscript included responses from pregnant women p4–p9. Interviews were conducted in person in Urdu with all participants who provided written informed consent and lasted 30–45 min in a private room at the PHC. To ensure rigor, two of the three researchers who were fluent in the local language were present at the time of each interview, with one conducting the interview and the other taking field notes. The initial interviews were conducted by LH, FA, and HJ, while NY, AK, and ZH provided supervision. Recordings were transcribed verbatim in Romanized Urdu and subsequently translated into English. For pregnant women, thematic saturation was achieved after six IDIs, defined as the point at which no new themes or meaningful insights emerged from successive interviews. For HCPs, all three POCUS-trained providers at the study site were interviewed, representing a complete census of the available provider pool rather than a sample drawn from a larger population; consequently, the concept of thematic saturation does not strictly apply to the HCP group, and findings for this group should be interpreted as a comprehensive within-site account rather than a saturated theme set. All transcripts were reviewed for completeness, anonymity, and clarity prior to coding and analysis. This study received approval from The Aga Khan University’s Ethics Review Committee (ERC # 2025-11503-35270).

### Data management and analysis

An inductive approach for thematic analysis was undertaken by the research team using interview transcripts and field notes. The process was guided by Braun and Clarke’s six-step framework, which included data familiarization, initial code generation, developing and refining themes, evaluating their relevance, and reporting the findings [13]. The research team consisted of female multidisciplinary health researchers (LH has just completed medical school while the rest of the team members had postgraduate education), all of whom had prior research experience and had attended training workshops in qualitative methods and thematic analysis. All team members except LH and FA were involved as investigators (ZH, FJ, IN, KR and JS), project management staff (NY, AK, BF) or data management for the FAMLI study (HJ, IF). Analyses were performed separately for pregnant women and HCPs. Audio recordings of all IDIs conducted in Urdu were first transcribed verbatim in MS Word and then translated into English by authors who are fluent in both languages. Two researchers who were present during each IDI subsequently conducted transcription and translation to maintain contextual accuracy and familiarity with the data. All transcripts were completed and reviewed by the research team to ensure qualitative rigor. Following data familiarization and confirmation that data saturation had been achieved, data were imported into Dedoose (version 9.2.22) [14]. Initial open coding was done by LH, FA, and HJ using sensitizing concepts derived from the interview guides, with senior review provided by NY, AK, and ZH. As the analysis evolved, codes were constantly compared, merged, and reorganized. Consistency in coding was ensured through regular consensus meetings, where discrepancies were discussed and resolved collaboratively. After thorough cross-checking, a final set of codes with relevant data extracts was formulated and grouped into major themes using MS Excel, aligned with study objectives. Final themes and representative quotations were agreed upon through team discussions and consensus. The study is reported in accordance with the Consolidated Criteria for Reporting Qualitative Research (COREQ) [15], with the completed 32-item checklist provided as a supplementary file (S3).

### Positionality statement

As a qualitative exploratory study, we gave careful attention to reflexivity and researcher positioning throughout the process. The research team included members at different career stages and with different levels of expertise. Data collection was carried out by three research staff (LH, FA, and HJ), each with varied clinical and research backgrounds, under the direct supervision of more experienced team members (NY and AK) and public health experts (ZH, IN, FJ, and JS). The three HCPs who participated as interviewees also differed in their background and experience: one was an expert sonographer with advanced clinical and medical expertise, while the other two were community-based health workers who had completed a one-time eight-hour POCUS training as part of the parent project’s training requirements, which covered device handling, probe placement, and basic identification of key anatomical positions. Competency was assessed through direct observation by the supervising expert sonographer before independent scanning began, and ongoing supervision and support were provided throughout the study period. These two health workers came from the same community as the pregnant women in the study. This range of perspectives among the HCP participants was noted and considered during analysis. The team discussed how their own assumptions, training, and prior experience might shape the way the interview was conducted and the data analyzed during the study planning, data collection and analysis phase. The team worked to stay grounded in what participants actually said, and coding decisions were reviewed regularly in group meetings. Any differences in how team members coded the data were discussed until a shared view was reached. Senior members (ZH, JS, IN, and FJ) reviewed the final set of themes to check that they were well-supported by the data and that no alternative interpretations had been overlooked.

The research staff who collected data (LH, FA, and HJ) were trained health professionals affiliated with the Aga Khan University and were not from the peri-urban community where the study took place. This meant there was a gap in lived experience between the data collectors and the participants. While all were from the same city, the differences in education, professional role, and social background meant the team approached the fieldwork as relative outsiders to the community’s day-to-day realities. The team was aware that their clinical and academic training could lead them to read participants’ accounts through a medical lens. To reduce this risk, they worked to treat participants’ own words as the starting point for analysis and to question their own initial interpretations during coding and team discussions.

The team’s affiliation with the Aga Khan University and, through it, the PHC, also shaped the research. This connection built trust and helped with community access because the university already had a presence in the area. At the same time, it reflected differences in social, economic, and professional status between the team and the participants. To reduce the influence of a mainly medical view of the data, the team made a deliberate effort to center participants’ own accounts and to treat them as the experts on their own lives.

## Results

A total of nine participants were included in this qualitative study, comprising six pregnant women and three healthcare providers. The pregnant women were predominantly multiparous and unemployed, with varied educational backgrounds. The healthcare providers represented mixed levels of training and ultrasound expertise, including one expert sonologist and two non-specialist users. Detailed demographic characteristics are presented in Table 1.

**Table 1:** Demographic characteristics of pregnant women and the healthcare providers

Pregnant Women (*N* = 6)	*n* (%)
**Age**,** years; median (range)**	29.5 (27–37)
**Parity; median (range)**	3.5 (1–4)
Primiparous	1 (16.7)
Multiparous	5 (83.3)
**Education**	
Primary or below	2 (33.3)
Secondary	2 (33.3)
Higher Secondary or above	2 (33.3)
**Occupation**	
Unemployed	6 (100)
**Healthcare Providers (N = 3)**	**n**
**Age**,** years; median (range)**	36 (30–43)
**Education**	
Higher Secondary or below	1
Graduate/Postgraduate	2

Thematic analysis was conducted separately for each participant group and common themes were identified. These included: role of ultrasound, perceived utility of POCUS, limitations and areas of improvement, myths and misconceptions regarding ultrasound, and recommendations for future use. Each participant was assigned a unique identifier to ensure confidentiality. Quotations are presented using participant IDs, where “HCP” denotes healthcare providers (HCP 01–03) and “PW” denotes pregnant women (PW 04–09).

### Theme 1: Role of ultrasound

HCPs and pregnant women shared that ultrasound plays a vital role in confirming pregnancy and accurately determining the gestational age. Pregnant women viewed it as a reliable way to confirm pregnancy, determine gestational age, and monitor the baby’s growth and well-being, which helped them feel reassured throughout their pregnancy.

*Ultrasound is important because we can see everything about the baby…how the brain is*,* how the hands are*,* how the kidneys are*,* how the legs are. Basically*,* it tells you everything. (PW 07)*

HCPs highlighted its importance in establishing accurate gestational dating, evaluating fetal growth, and enabling early detection of complications that support timely therapeutic decisions and improve outcomes.

*The role of ultrasound is that we can see whether the baby is healthy or not. We look at every part of the baby*,* from head to toe…We also check whether the gestational age matches the baby’s growth*,* if the baby is growing according to the expected timeline. (HCP 03)*

### Theme 2: Perceived utility of POCUS

Both pregnant women and HCPs reported their experiences with POCUS as positive and emphasized its value for ANC. Many appreciated the quick and convenient nature of the scan. While a few women reported initial anxiety when encountering the device for the first time, most described feeling comfortable once the procedure and its purpose were explained.

*I don’t know*,* it just felt strange… like*,* what kind of machine is this? Then the lady told me that it’s fine*,* don’t be worried*,* nothing will happen…it’s also an ultrasound. (PW 06)*

HCPs described POCUS as an efficient and convenient adjunct to routine ultrasound in ANC. Providers emphasized that it allows for immediate bedside assessments of fetal status and could support rapid clinical decision-making.

*POCUS in my opinion is just like a commercial machine*,* I don’t think there is much difference between POCUS and a commercial ultrasound (HCP 02)*

Pregnant women described a range of advantages associated with the use of POCUS in ANC such as its ease of use, and ability to provide rapid results without prolonged waiting. They also indicated that the portable nature of the device could reduce their need for travel to health facilities.

*Yes*,* it’s small*,* easy to use*,* gets done quickly…for the big machine…you have to come to the center yourself (PW 06)*

HCPs also appreciated the portability and ease of use of the device.

*The ease comes from its simple interface*,* it’s very user-friendly (HCP 02)*

Another participant described:

*If a patient has an emergency*,* we can quickly go to her home and scan her. We can check for the baby’s heartbeat and movements*,* to see if the baby is okay or not. (HCP 03)*

Together, these accounts point toward the ease of use of POCUS, while both PWs and HCPs could envision the future benefit of home-based scanning where access to healthcare may be challenging.

### Theme 3: Limitations and areas of improvement

Despite its advantages, both HCPs and pregnant women acknowledged certain limitations of POCUS that influenced its use in ANC. Pregnant women provided relatively limited reflections on drawbacks, with most views relating to a preference for conventional ultrasound, often shaped by prior experience and the perception that a full, formal scan provides greater diagnostic assurance.

*No*,* this one (points to POCUS) is a different thing*,* that one is a different thing. The big machine seems better. We’ve always had it done with that (conventional ultrasound). (PW 06)*

*The difference mainly lies in the resolution. Obviously*,* the larger commercial machines have much better resolution and more advanced settings for image clarity. (HCP 02)*

While HCPs valued the clinical utility of POCUS, they acknowledged that it may be less suitable for detecting complex abnormalities or providing detailed imaging compared with conventional ultrasound.

### Theme 4: Myths and misconceptions regarding ultrasound

Although not directly related to POCUS, pregnant women described myths and misconceptions related to the use of ultrasound during pregnancy, including concerns about potential harm from repeated scans.

*They said that getting ultrasounds done repeatedly is discouraged. Like*,* if we get it done 2–3 times in a month*,* then the baby is exposed to radiation*,* and it can cause problems in the eyes (PW 07)*

HCPs also shared insights into how POCUS was associated with fear and misconceptions among some women.

*Yes*,* sometimes some patients get scared. They worry that since this is a “different” ultrasound*,* maybe it could harm them or the baby. (HCP 03)*

### Theme 5: Recommendations for future use

Pregnant women and HCPs described strong support for the continued use of POCUS in ANC.

*Yes*,* absolutely*,* I will tell them. ….I’ll go and inform them to come to the center and get the scan with POCUS. (PW 04)*

HCPs discussed the adoption of POCUS into routine ANC, reflecting on both facilitators and barriers to its use.

*If they have difficulty reaching the facility or any kind of obstacle*,* we could go to their homes and do the scan*,* that would be excellent. (HCP 01)*

HCPs also emphasized the importance of educating women and families about the purpose, safety, and benefits of POCUS as a means to address misconceptions and build trust within the community.

*By holding sessions*,* big sessions*,* and going into communities to tell people about it. That way*,* people will have more information*,* and it will benefit us a lot too. (HCP 03)*

The long-term sustainability of POCUS in ANC was dependent on the availability of reliable technical support and continuous capacity building.

*But if you want proper implementation*,* then actual training programs will have to be organized. (HCP 02)*

Table 2 summarizes five overarching themes identified across both participant groups and mapped to the corresponding TAM constructs. HCPs and PWs largely viewed POCUS as a useful and accessible tool for antenatal care, valuing its portability, ease of use, and ability to provide rapid fetal assessment. Acceptance was affected by persistent myths, particularly fears of radiation harm from repeated scans, and by a preference for conventional ultrasound among some women. HCPs identified the need for structured training and community education as prerequisites for wider scale-up.

**Table 2:** Summary of themes, key content, and representative quotes by participant group

Theme	Sub-theme	Representative Quote	Participant Group	TAM Construct
Role of Ultrasound	Enhancing antenatal care	“The role of ultrasound is that we can see whether the baby is healthy or not. We look at every part of the baby, from head to toe…We also check whether the gestational age matches the baby’s growth, if the baby is growing according to the expected timeline.” (HCP 03)	HCP	Perceived Usefulness
	Improving access to ultrasound	“If they have difficulty reaching the facility or any kind of obstacle, we could go to their homes and do the scan, that would be excellent.” (HCP 01)	HCP	Perceived Usefulness
Perceived Utility of POCUS	Portability and rapid assessment	“If a patient has an emergency, we can quickly go to her home and scan her. We can check for the baby’s heartbeat and movements, to see if the baby is okay or not.” (HCP 03)	HCP	Perceived Usefulness
	Reassurance and visualization of the fetus	“Ultrasound is important because we can see everything about the baby…how the brain is, how the hands are, how the kidneys are, how the legs are. Basically, it tells you everything.” (PW 07)	PW	Perceived Usefulness
	User-friendly interface	“The ease comes from its simple interface, it’s very user-friendly.” (HCP 02)	HCP	Perceived Ease of Use
Myths and Misconceptions Regarding Ultrasound	Fear of harm to the fetus	“They said that getting ultrasounds done repeatedly is discouraged. Like, if we get it done 2–3 times in a month, then the baby is exposed to radiation, and it can cause problems in the eyes.” (PW 07)	PW	External Factors Influencing Acceptance
	Concerns about unfamiliar technology	“I don’t know, it just felt strange…like, what kind of machine is this? Then the lady told me that it’s fine, don’t be worried, nothing will happen…it’s also an ultrasound.” (PW 06)	PW	External Factors Influencing Acceptance
Limitations and Areas of Improvement	Technical and diagnostic limitations	“The difference mainly lies in the resolution. Obviously, the larger commercial machines have much better resolution and more advanced settings for image clarity.” (HCP 02)	HCP	External Factors Influencing Acceptance
	Need for additional training	“But if you want proper implementation, then actual training programs will have to be organized.” (HCP 02)	HCP	External Factors Influencing Acceptance
Recommendations for Future Use	Willingness to recommend and scale-up	“Yes, absolutely, I will tell them. I’ll go and inform them to come to the center and get the scan with POCUS.” (PW 04)	PW	Behavioral Intention
	Integration into routine services	“By holding sessions, big sessions, and going into communities to tell people about it. That way, people will have more information, and it will benefit us a lot too.” (HCP 03)	HCP	Behavioral Intention

## Discussion

This qualitative study explored the perceptions of POCUS among HCPs and pregnant women in a peri-urban community in Karachi. Overall, POCUS was perceived as a feasible and acceptable tool for antenatal care, primarily because of its portability and utility in settings where access to conventional imaging is limited. Participants valued its ability to provide on-site assessment but also identified community- and system-level barriers that need to be addressed to ensure broader integration in routine ANC services in LMICs.

Overall, the general perception from pregnant women and HCPs regarding POCUS was quite positive. Among pregnant women, satisfaction with POCUS was driven by real-time direct visualization of the fetus during brief scans, which helped build trust in the care received. This finding aligns with previous literature where women described antenatal POCUS as reassuring, valued provider explanations regarding fetal well-being, and expressed willingness to recommend this service to others [16]. Balmuth et al., also observed high patient satisfaction in hospital settings, with patients perceiving shorter scan durations, highlighting how rapid point-of-care imaging may enhance the perceptions of care efficiency [17]. HCPs also viewed POCUS as beneficial for supporting timely clinical decision-making during ANC. Consistent with evidence from both high-income countries and LMICs, nurses and midwives have reported antenatal POCUS to be useful for assessing key obstetric parameters, including fetal number and presentation, and for guiding referral decisions and maternal counselling [18, 19].

The findings from this study can be viewed in the context of the Technology Acceptance Model (TAM); both pregnant women and healthcare providers perceived POCUS as useful for the rapid visualization and assessment of fetal well-being, reflecting high perceived usefulness, a key construct of the TAM. HCPs also noted the device’s portability and user-friendly interface but acknowledged the need for regular training to ensure adoption. These findings are in line with other studies where constructs described in the TAM play a critical role in the adoption and scalability of healthcare technologies in many world regions [20].

The perceived value of POCUS among both groups was intricately linked to convenience and time efficiency, particularly when services are delivered closer to communities. Similar findings have been reported from Kenya, where women valued reduced travel and waiting times as well as the potential for lower out-of-pocket expenses, thereby reducing delays in accessing obstetric imaging [21, 22]. Qualitative research from rural Nepal reported that trained skilled birth attendants perceived decentralized obstetric ultrasound services and mobile outreach as beneficial for improving access to ANC, enabling earlier identification of high-risk cases, and supporting timely assessments closer to women’s homes [23]. A mixed-methods study of HCPs from Africa also reported that > 75% of providers believed that POCUS would improve ANC attendance and clinical decision-making but needed a strong referral pathway to have a meaningful impact on clinical outcomes [24]. Taken together, these findings indicate that the acceptability of POCUS is shaped by its ability to ease access barriers for pregnant women, while functioning as an efficient clinical tool for HCPs.

Despite its benefits, POCUS uptake is limited by user- and system-level barriers. Evidence from sub-Saharan Africa similarly found that POCUS in ANC was initially viewed as modern but unfamiliar and raised concerns about potential maternal or fetal harm [21, 22]. As reflected in our findings, HCPs perceived the limitations of POCUS in obstetrics as primarily technical (e.g., reduced resolution for complex abnormalities) and operational (e.g., ongoing training and technical support needs). Additional studies in similar LMIC contexts have also highlighted challenges such as charging difficulties and unreliable electricity [25, 26]. Lower image resolution also shaped expectations around its diagnostic scope. This perception is supported by comparative evidence showing conventional ultrasound machines produce higher image resolution and overall image quality than handheld POCUS devices, particularly in obstetric scanning [27]. Nevertheless, POCUS was valued for its user-friendly design and short learning curve, with portability seen as a key advantage in emergency situations where women may be unable to reach healthcare facilities [27].

To leverage the advantages offered by POCUS, investments should prioritize building community trust in the tool and implementing structured training programs to support task shifting to frontline care providers. Evidence from Kenya demonstrates that large-scale training initiatives have expanded the number of rural HCPs able to perform obstetric POCUS, thereby increasing service capacity and coverage for pregnant women [28]. Synergized efforts for task shifting for POCUS in LMICs have been associated with improved identification of high-risk pregnancies and strengthened referral pathways, ultimately contributing to improved patient outcomes [25].

To the best of our knowledge, this is among the first qualitative studies in Pakistan to explore perceptions of obstetric POCUS from both HCPs and pregnant women, providing a dual perspective on acceptability and feasibility from an LMIC. To avoid any recall bias and account for the low education levels, women who had a recent POCUS and conventional scan were recruited. Despite this strength, study limitations must be acknowledged. The study was conducted in a single peri-urban setting where the parent study was conducted, which may limit its generalizability. In addition, only three HCPs were included, which may not fully capture the range of provider experiences that could emerge during broader implementation of POCUS. Within this small group, one was an expert sonographer with advanced clinical expertise, and it is possible that this individual’s views disproportionately shaped the HCP findings. In larger-scale POCUS programs, the provider workforce would include nurses, midwives, physicians, and community health workers, including female community health workers with lower levels of formal training, whose perspectives and experiences may differ substantially from those represented here. Since participants were recruited from an ongoing parent study in which they received POCUS from the same institution conducting the interviews, the findings may be subject to social desirability bias and selection bias. Participants were already enrolled in an ongoing POCUS study and had been exposed to the device prior to interview; those with negative views may have been less likely to volunteer, and participants who remained in the cohort may represent those with more favorable attitudes toward the intervention. Participants who declined to join the study, or who have never encountered POCUS, may hold substantially different views. Although participants were explicitly assured that their responses would have no bearing on their continued enrolment in the parent cohort or the care they receive at the facility, these factors may have influenced their responses. Finally, because eligible women were required to have undergone a recent POCUS scan, the findings reflect the perceptions of women already exposed to POCUS and may not capture the views of POCUS-naïve women, whose acceptability may differ.

## Conclusion

This study suggests that obstetric POCUS is both acceptable and feasible in a peri-urban LMIC setting when integrated within existing antenatal care services. Its portability and ability to provide real-time reassurance may reduce access barriers and support timely referral, particularly where conventional imaging is limited. However, successful scale-up will require structured training, ongoing technical support, community engagement to address misconceptions, and strong referral linkages. With appropriate health system investment, POCUS has the potential to expand equitable access to essential obstetric imaging, support timely clinical decision-making, and strengthen community-level ANC in underserved settings, though prospective outcome studies are needed to confirm impact on maternal and neonatal outcomes.

## Supplementary Information

Below is the link to the electronic supplementary material.

**Supplementary Material 1: **S1_IDI Guide for pregnant women

**Supplementary Material 2:** S2_IDI Guide for HCPs

**Supplementary Material 3:** S3_COREQ

## Data Availability

The datasets used during the current study are available from the corresponding author on reasonable request.

## Abbreviations

ANC: Antenatal Care
LMIC: Low- and Middle-Income Countries
POCUS: Point-Of-Care Ultrasound
HCP: Healthcare Provider
PW: Pregnant Woman
GA: Gestational Age
ICF: Informed consent form
WHO: World Health Organization
PHC: Primary Health Center
CHW: Community Health Worker
FAMLI: Fetal Age Machine Learning Initiative
IDI: In-Depth Interview

## References

[1] Huang K, Tao F, Raven J, Liu L, Wu X, Tang S. Utilization of antenatal ultrasound scan and implications for caesarean section: a cross-sectional study in rural Eastern China. BMC Health Serv Res. 2012;12:93. 10.1186/1472-6963-12-93. 22494358 10.1186/1472-6963-12-93 PMC3350450

[2] World Health Organization. Imaging ultrasound before 24 weeks of pregnancy: 2022 update to the WHO antenatal care recommendations for a positive pregnancy experience. Geneva: WHO; 2022. 35442602

[3] Shah S, Bellows BA, Adedipe AA, et al. Perceived barriers in the use of ultrasound in developing countries. Crit Ultrasound J. 2015;7:11. 10.1186/s13089-015-0028-2. 26123609 10.1186/s13089-015-0028-2 PMC4485671

[4] Fraleigh CD, Duff E. Point-of-care ultrasound: An emerging clinical tool to enhance physical assessment. Nurse Pract. 2022;47(8):14–20. 10.1097/01.NPR.0000841944.00536.b2. 35877142 10.1097/01.NPR.0000841944.00536.b2 PMC9301985

[5] Recker F, Weber E, Strizek B, et al. Point-of-care ultrasound in obstetrics and gynecology. Arch Gynecol Obstet. 2021;303(4):871–6. 10.1007/s00404-021-05972-5. 33558990 10.1007/s00404-021-05972-5 PMC7985120

[6] Dalmacion GV, Reyles RT, Habana AE, et al. Handheld ultrasound to avert maternal and neonatal deaths in 2 regions of the Philippines: an iBuntis intervention study. BMC Pregnancy Childbirth. 2018;18:32. 10.1186/s12884-018-1658-8. 29347926 10.1186/s12884-018-1658-8 PMC5774122

[7] Wanjiku GW, Bell G, Kapadia S, et al. Impact of point-of-care ultrasound use on patient referral decisions in rural Kenya: a mixed methods study. BMC Health Serv Res. 2024;24:212. 10.1186/s12913-024-10673-1. 38360660 10.1186/s12913-024-10673-1 PMC10870490

[8] Kimberly HH, Murray A, Mennicke M, et al. Focused maternal ultrasound by midwives in rural Zambia. Ultrasound Med Biol. 2010;36(8):1267–72. 10.1016/j.ultrasmedbio.2010.05.017. 20691916 10.1016/j.ultrasmedbio.2010.05.017

[9] Schoch M, Bennett PN, Currey J, et al. Nurses’ perceptions of point-of-care ultrasound for haemodialysis access assessment and guided cannulation: a qualitative study. J Clin Nurs. 2023;32(23–24):8116–25. 10.1111/jocn.16877. 37661364 10.1111/jocn.16877

[10] Onsongo LN, Bett SC, Gachuiri GW, et al. Perspectives of health care providers on obstetric point-of-care ultrasound in lower-level health facilities in Kenya. Midwifery. 2025. 10.1016/j.midw.2024.104196. 39357458 10.1016/j.midw.2024.104196 PMC11619753

[11] Muhammad Zeb MI, Shahid S, Naeem K, Fatima U, et al. Profile: maternal and child health surveillance system in Peri-urban areas of Karachi, Pakistan. Gates Open Res. 2022;2:2.

[12] King WR, He J. A meta-analysis of the technology acceptance model. Inf Manag. 2006;43(6):740–55. 10.1016/j.im.2006.05.003.

[13] Cooper HE, Camic PM, Long DL, et al. APA handbook of research methods in psychology. Volume 2. Washington DC: American Psychological Association; 2012.

[14] Lieber E, Salmona M, Kaczynski D. Introduction to Dedoose for mixed analysis. In: Routledge reviewer’s guide to mixed methods analysis. Routledge; 2021. pp. 319–30.

[15] Tong A, Sainsbury P, Craig J. Consolidated criteria for reporting qualitative research (COREQ): a 32-item checklist for interviews and focus groups. Int J Qual Health Care. 2007;19(6):349–57. 10.1093/intqhc/mzm042. 17872937 10.1093/intqhc/mzm042

[16] Payesa C, Seyama L, Chimwaza Y, et al. Feasibility and acceptability of point-of-care ultrasound delivered by midwives during routine antenatal care in Malawi. BMJ Open. 2025;15:e100515. 10.1136/bmjopen-2025-100515. 40784784 10.1136/bmjopen-2025-100515 PMC12336590

[17] Balmuth EA, Luan D, Jannat-Khah D, et al. Point-of-care ultrasound (POCUS): Assessing patient satisfaction and socioemotional benefits in hospital setting. PLoS ONE. 2024;19:e0298665. 10.1371/journal.pone.0298665. 38363766 10.1371/journal.pone.0298665 PMC10871481

[18] Mubuuke AG, Erem G, Nassanga R, et al. Point-of-care obstetric ultrasound training for midwives and nurses in Sub-Saharan Africa: qualitative study. BMC Res Notes. 2023;16:287. 10.1186/s13104-023-06569-8. 37875986 10.1186/s13104-023-06569-8 PMC10598935

[19] Johnston BK, Darling EK, Malott A, et al. Canadian midwives’ perspectives on point-of-care ultrasound in obstetrical care. Heliyon. 2024;10(6). 10.1016/j.heliyon.2024.e27512. 10.1016/j.heliyon.2024.e27512 PMC10963237 38533003

[20] AlQudah AA, Al-Emran M, Shaalan K. Technology acceptance in healthcare: a systematic review. Appl Sci. 2021;11(22):10537. 10.3390/app112210537.

[21] Kumar MB, Mulongo CM, Pincerato L, et al. Task sharing for increasing access to obstetric ultrasonography: a formative qualitative study of nurse-led scanning with telemedicine review in Kenya. Oxf Open Digit Health. 2024;2:oqae037. 10.1093/oodh/oqae037. 40230981 10.1093/oodh/oqae037 PMC11932403

[22] Koech A, Musitia PM, Mwashigadi GM, et al. Acceptability and feasibility of a low-cost device for gestational age assessment in a low-resource setting. JMIR Hum Factors. 2022;9:e34823. 10.2196/34823. 36574278 10.2196/34823 PMC9832351

[23] Kim C, Wagle K, Shrestha B, et al. Perceptions of service providers, service recipients and female community health volunteers on a rural obstetric ultrasound program in rural Nepal: a qualitative study. BMC Pregnancy Childbirth. 2023;23:574. 10.1186/s12884-023-05876-z. 37563558 10.1186/s12884-023-05876-z PMC10413490

[24] Della Ripa S, Santos N, Walker D. AI-enabled obstetric point-of-care ultrasound in low- and middle-income countries. BMC Pregnancy Childbirth. 2025;25:1–17. 10.1186/s12884-025-07796-6. 40615993 10.1186/s12884-025-07796-6 PMC12229288

[25] Abrokwa SK, Ruby LC, Heuvelings CC, et al. Task shifting for point-of-care ultrasound in low- and middle-income countries: systematic review. EClinicalMedicine. 2022;45:101333. 10.1016/j.eclinm.2022.101333. 35284806 10.1016/j.eclinm.2022.101333 PMC8904233

[26] Bellows B, Totten J, Shah S, et al. Perceived barriers in point-of-care ultrasound use in WWAMI region. J Emerg Med Crit Care. 2015;1:4.

[27] Salimi N, Gonzalez-Fiol A, Yanez ND, et al. Ultrasound image quality comparison between handheld and mid-range ultrasound machine. POCUS J. 2022;7:154. 10.24908/pocus.v7i1.15052. 36896280 10.24908/pocus.v7i1.15052 PMC9979954

[28] Githemo G, Wanyoro A, Masika J, et al. Evaluation of large-scale implementation of obstetric point of care ultrasound in eight counties in Kenya using RE-AIM framework. BMC Health Serv Res. 2025;25(1):1016. 10.1186/s12913-025-13212-8. 40751247 10.1186/s12913-025-13212-8 PMC12315356

