# Supplementary material for: Providers’ and pregnant women’s perspectives on obstetric point-of-care ultrasound use in peri-urban Karachi, Pakistan: a qualitative study
Source: BMC Digit Health. 2026 Aug 20;4(1):64. doi: 10.1186/s44247-026-00295-4 (PMC13493394; doi:10.1186/s44247-026-00295-4)
Supplement: Supplementary file 1 [file 44247_2026_295_MOESM1_ESM.pdf]

<u>Title:</u> Perception of obstetric point-of-care ultrasound among healthcare providers and pregnant women in peri-urban communities of Karachi, Pakistan

IDI Guide for Healthcare Providers

My name is XXX, and I am working as a Research Fellow at XXX. I am grateful to you for taking out time to take part in this interview. Today, I will be asking some questions regarding your perception of point-of-care ultrasound (POCUS) in antenatal care. We are specifically interested in exploring your experience with using POCUS as one of the trained staff for the FAMLI cohort. Through this interview, we hope to gain insight regarding the experiences with using POCUS, its advantages and disadvantages and how we can develop effective strategies in improving its adoption and enhancing antenatal care. There is no “right” or “wrong” answers to the questions I ask you. If any of the questions asked are unclear, please let me know and I can explain. If there are any questions that you do not wish to answer, please say so and I will move to the next question. We will be audio-recording this interview, and I will also be taking notes. Do you have any questions before we begin?

Data Collection Date ________________________ Start time/End time____________________________

IDI Location ________________________________

Interviewer____________________________________

Notetaker____________________________________

Demographics:

1. What is your age?
2. What is the highest degree or level of education you have completed?
3. What is your current employment status/How long have you been working at this healthcare facility?
4. What is the nature of your work at this facility?
5. Where were you trained in POCUS? How long was the training for? (Please tell specific time and duration)
6. Approximately how many patients do you perform POCUS on daily basis? How long have you been doing this for?

Interview Prompts

Opening Question:

- Can you describe the general routine antenatal care that is provided to pregnant woman at this health facility?
  Probes: what services are included? How often do pregnant women avail these? How many women come to this facility daily? What are the women's perceptions of this facility?

- What role do you think ultrasound plays in antenatal care and how does it influence clinical decision making?

- Can you describe your overall experience with POCUS in your work?
  Probes: how did you feel when you first started? Was it difficult or easy to use? How has your confidence and comfort changed over time? If you compare its use with routine ultrasound, what differences have you observed?

- What do you like about POCUS? and why?
  Probes: what advantages does it offer compared to the conventional machine? Can it be done in Less amount of time? It's small? It's easy to carry?

- What do you not like about using POCUS? How do you think we can improve that?

- While performing POCUS, have you ever encountered any significant finding? (for expert user)
  Probes: how did you respond to it? Whenever you come across a finding that is out of the ordinary, what is the course of action? Are the findings documented somewhere?

- What are your thoughts regarding incorporating POCUS in routine antenatal care?
  Probes: who do you think should perform it, doctor, nurse, sonologist? in your opinion, what are pregnant women's perceptions of it?

- How do you feel about clinic staff performing POCUS during clinic visits?
  Probes: what do you think about the time needed to perform it? What do you think about the space needed to perform it? would it interfere with the routine functioning of the clinic? Will it have any benefit?

- What do you think would be the biggest challenge in incorporating POCUS in clinics? *Probes: will it prolong the clinic duration? long queues?*

- How to do you think pregnant women in your community perceived POCUS? *Probes: do they accept it? Any misconception or fear?*

- To what extent, do you think, POCUS can be used to manage pregnant women antenatally? *Probes: what can it be used for? can it be helpful for clinical decision making? If you compare it with conventional ultrasound, what aspects might differ? (expert user)*

- Based on your experience what would you suggest using POCUS more effectively/better in your setting?

- What structures/strategies should be put in place to enhance the use of POCUS in your daily practice?
  Probes: will enhanced supervision, workshops, structured training and technical support encourage healthcare workers to adopt POCUS?

Closing Question:

- Is there anything else we haven't covered that you thought we would talk about?

Thank you so much for taking out time to participate in this interview today.
