# Supplementary material for: Providers’ and pregnant women’s perspectives on obstetric point-of-care ultrasound use in peri-urban Karachi, Pakistan: a qualitative study
Source: BMC Digit Health. 2026 Aug 20;4(1):64. doi: 10.1186/s44247-026-00295-4 (PMC13493394; doi:10.1186/s44247-026-00295-4)
Supplement: Supplementary file 2 [file 44247_2026_295_MOESM2_ESM.pdf]

Title: Perception of obstetric point-of-care ultrasound (POCUS) among healthcare providers and pregnant women in peri-urban communities of Karachi, Pakistan

IDI Guide for Pregnant Women

My name is XXX, and I am working as a Research Fellow at XXX. Thank you for taking out time to take part in this interview. Today, I will be asking some questions regarding your perception of point-of-care ultrasound (POCUS). We are specifically interested in exploring your experience with POCUS while being enrolled in the FAMLI cohort. Through this interview, we hope to gain insight regarding your experiences with POCUS, its advantages and disadvantages, and this will help us improve its adoption and enhance antenatal care. There is no “right” or “wrong” answers to the questions I ask you. If any of the questions asked are unclear, please let me know and I will repeat them. If there are any questions that you do not wish to answer, please say so and I will move to the next question. We will be audio-recording this interview, and I will also be taking notes. Do you have any questions before we begin?

Data Collection Date ________________________

Start time/End time____________________________

IDI Location ________________________________

Interviewer___________________________________

Notetaker____________________________________

Demographics:

1. What is your age?
2. Gravidity and Parity (will be noted from the medical record)
3. What is your husband’s occupation?
4. What is the highest level of education you have attained?
5. What is your current occupation?
6. Is this your first time coming to this health facility? Is this your first visit during this pregnancy?

Interview Prompts

Opening Question:

- What are your general thoughts about maternal and child health?
  Probes: how important is it for a pregnant woman to receive antenatal care? what factors encourage women to go to these health facilities? has any woman in your family or neighborhood experienced complications during pregnancy or childbirth?

- In your opinion, why is ultrasound important?
  Probes: explore the reasons and perceptions about ultrasound (e.g., to confirm pregnancy, to know GA, baby growth, etc.)

- How accessible is it for you to get an ultrasound scan done?
  Probes: mode of transport? do you go alone? cost of travel? how long does it take you? where is most of the time spent? are there any challenges you face?

- Are there any myths and misconceptions in your community about using ultrasound during pregnancy?
  Probes: has anyone ever discouraged you from getting an ultrasound? why? has anyone ever encouraged you to get an ultrasound done?

- Can you describe your experience with POCUS during this pregnancy?
  Probes: what do you like about it? was there anything you did not like? were you feeling comfortable or nervous? did it cause any pain or discomfort?

- How did you feel when POCUS was placed on you?
  Probes: did it make you feel anxious? did you feel you were getting better or worse care?

- Is there anything you would like to share about POCUS that is different from conventional ultrasounds?
  Probes: time taken? information received?
  **Note: only for those who had a previous scan.*

- What do think are the advantages of using POCUS during pregnancy? *Probes: more accessible? quicker diagnosis? less time?*

- What do you think are the disadvantages of using POCUS during pregnancy? *Probes: inaccuracy of results? Less information received?*

- If your healthcare workers give you important information by conducting POCUS regarding the pregnancy and fetus, would you trust those results? Why or why not?
  Probes: Why is it trustworthy? Why is it not trustworthy? Would you still want the conventional scan?

- Would you opt for POCUS in future pregnancies? Why or why not?

- Would you recommend it to other pregnant women? Why or why not? *Probes: what will you tell them regarding POCUS?*

Closing Question:

- Is there anything else we haven't covered that you thought we would talk about?

Thank you so much for taking out time to participate in this interview today.
